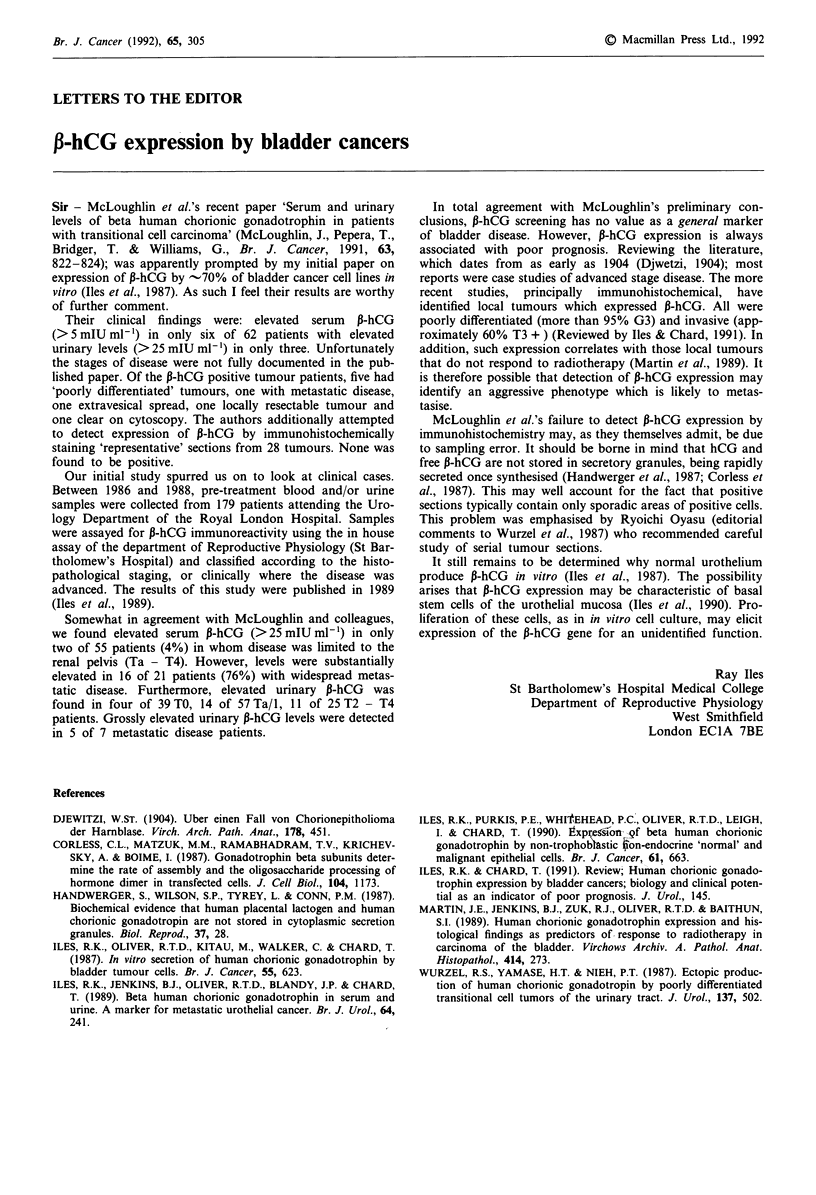# Beta-hCG expression by bladder cancers.

**DOI:** 10.1038/bjc.1992.62

**Published:** 1992-02

**Authors:** R. Iles


© Macmillan Press Ltd., 1992

### LETTERS TO THE EDITOR

### β-hCG expression by bladder cancers

**Sir** – McLoughlin *et al.*'s recent paper 'Serum and urinary levels of beta human chorionic gonadotrophin in patients with transitional cell carcinoma' (McLoughlin, J., Pepera, T., Bridger, T. & Williams, G., *Br. J. Cancer*, 1991, **63**, 822–824); was apparently prompted by my initial paper on expression of β-hCG by ~70% of bladder cancer cell lines *in vitro* (Iles *et al.*, 1987). As such I feel their results are worthy of further comment.

Their clinical findings were: elevated serum β-hCG (>5 mIU $ml^{-1}$) in only six of 62 patients with elevated urinary levels (>25 mIU $ml^{-1}$) in only three. Unfortunately the stages of disease were not fully documented in the published paper. Of the β-hCG positive tumour patients, five had 'poorly differentiated' tumours, one with metastatic disease, one extravesical spread, one locally resectable tumour and one clear on cytoscopy. The authors additionally attempted to detect expression of β-hCG by immunohistochemically staining 'representative' sections from 28 tumours. None was found to be positive.

Our initial study spurred us on to look at clinical cases. Between 1986 and 1988, pre-treatment blood and/or urine samples were collected from 179 patients attending the Urology Department of the Royal London Hospital. Samples were assayed for β-hCG immunoreactivity using the in house assay of the department of Reproductive Physiology (St Bartholomew's Hospital) and classified according to the histopathological staging, or clinically where the disease was advanced. The results of this study were published in 1989 (Iles *et al.*, 1989).

Somewhat in agreement with McLoughlin and colleagues, we found elevated serum β-hCG (>25 mIU $ml^{-1}$) in only two of 55 patients (4%) in whom disease was limited to the renal pelvis (Ta – T4). However, levels were substantially elevated in 16 of 21 patients (76%) with widespread metastatic disease. Furthermore, elevated urinary β-hCG was found in four of 39 T0, 14 of 57 Ta/1, 11 of 25 T2 – T4 patients. Grossly elevated urinary β-hCG levels were detected in 5 of 7 metastatic disease patients.

In total agreement with McLoughlin's preliminary conclusions, β-hCG screening has no value as a *general* marker of bladder disease. However, β-hCG expression is always associated with poor prognosis. Reviewing the literature, which dates from as early as 1904 (Djwetzi, 1904); most reports were case studies of advanced stage disease. The more recent studies, principally immunohistochemical, have identified local tumours which expressed β-hCG. All were poorly differentiated (more than 95% G3) and invasive (approximately 60% T3 + ) (Reviewed by Iles & Chard, 1991). In addition, such expression correlates with those local tumours that do not respond to radiotherapy (Martin *et al.*, 1989). It is therefore possible that detection of β-hCG expression may identify an aggressive phenotype which is likely to metastasise.

McLoughlin *et al.*'s failure to detect β-hCG expression by immunohistochemistry may, as they themselves admit, be due to sampling error. It should be borne in mind that hCG and free β-hCG are not stored in secretory granules, being rapidly secreted once synthesised (Handwerger *et al.*, 1987; Corless *et al.*, 1987). This may well account for the fact that positive sections typically contain only sporadic areas of positive cells. This problem was emphasised by Ryoichi Oyasu (editorial comments to Wurzel *et al.*, 1987) who recommended careful study of serial tumour sections.

It still remains to be determined why normal urothelium produce β-hCG *in vitro* (Iles *et al.*, 1987). The possibility arises that β-hCG expression may be characteristic of basal stem cells of the urothelial mucosa (Iles *et al.*, 1990). Proliferation of these cells, as in *in vitro* cell culture, may elicit expression of the β-hCG gene for an unidentified function.

Ray Iles
St Bartholomew's Hospital Medical College
Department of Reproductive Physiology
West Smithfield
London EC1A 7BE

### References

DJEWITZI, W.ST. (1904). Uber einen Fall von Chorionepitholioma der Harnblase. *Virch. Arch. Path. Anat.*, **178**, 451.

CORLESS, C.L., MATZUK, M.M., RAMABHADRAM, T.V., KRICHEVSKY, A. & BOIME, I. (1987). Gonadotrophin beta subunits determine the rate of assembly and the oligosaccharide processing of hormone dimer in transfected cells. *J. Cell Biol.*, **104**, 1173.

HANDWERGER, S., WILSON, S.P., TYREY, L. & CONN, P.M. (1987). Biochemical evidence that human placental lactogen and human chorionic gonadotropin are not stored in cytoplasmic secretion granules. *Biol. Reprod.*, **37**, 28.

ILES, R.K., OLIVER, R.T.D., KITAU, M., WALKER, C. & CHARD, T. (1987). *In vitro* secretion of human chorionic gonadotrophin by bladder tumour cells. *Br. J. Cancer*, **55**, 623.

ILES, R.K., JENKINS, B.J., OLIVER, R.T.D., BLANDY, J.P. & CHARD, T. (1989). Beta human chorionic gonadotrophin in serum and urine. A marker for metastatic urothelial cancer. *Br. J. Urol.*, **64**, 241.

ILES, R.K., PURKIS, P.E., WHITEHEAD, P.C., OLIVER, R.T.D., LEIGH, I. & CHARD, T. (1990). Expression of beta human chorionic gonadotrophin by non-trophoblastic non-endocrine 'normal' and malignant epithelial cells. *Br. J. Cancer*, **61**, 663.

ILES, R.K. & CHARD, T. (1991). Review; Human chorionic gonadotrophin expression by bladder cancers; biology and clinical potential as an indicator of poor prognosis. *J. Urol.*, 145.

MARTIN, J.E., JENKINS, B.J., ZUK, R.J., OLIVER, R.T.D. & BAITHUN, S.I. (1989). Human chorionic gonadotrophin expression and histological findings as predictors of response to radiotherapy in carcinoma of the bladder. *Virchows Archiv. A. Pathol. Anat. Histopathol.*, **414**, 273.

WURZEL, R.S., YAMASE, H.T. & NIEH, P.T. (1987). Ectopic production of human chorionic gonadotropin by poorly differentiated transitional cell tumors of the urinary tract. *J. Urol.*, **137**, 502.